# Unraveling the immune system’s role in peripheral nerve regeneration: a pathway to enhanced healing

**DOI:** 10.3389/fimmu.2025.1540199

**Published:** 2025-02-21

**Authors:** Xu Zhang, Yanxian Zhang, Yuqing Chen, Yuxiang Ji, Yongmei Lyu, Zengli Miao, Xuchu Duan, Xiaoyu Liu

**Affiliations:** ^1^ Key Laboratory of Neuroregeneration of Jiangsu and Ministry of Education, Co-innovation Center of Neuroregeneration; Department of Pharmacy, Affiliated Hospital of Nantong University; School of Life Science, Nantong Laboratory of Development and Diseases; Medical School, Nantong University, Nantong, China; ^2^ Clinical Medical Research Center, Department of Neurosurgery, Affiliated Wuxi Clinical College of Nantong University, Wuxi No.2 Peolpe’s Hospital, Jiangnan University Medical Center, Wuxi, China; ^3^ School of Marine and Bioengineering, Yancheng Institute of Technology, Yancheng, China

**Keywords:** immunity, peripheral nerve injury, peripheral nerve regeneration, immune cells, signaling pathway, immunoactive substances

## Abstract

Peripheral nerve injury (PNI) represents a common challenge in clinical practice. In contrast to the central nervous system (CNS), the peripheral nervous system (PNS) in mature mammals possesses a limited regenerative capacity. Upon the occurrence of PNI, peripheral nerve regeneration (PNR) is initiated, facilitated by the activation of the immune microenvironment and the intrinsic growth potential of neurons. This regenerative process encompasses several key stages, including distal axon degeneration, myelin breakdown, clearance of myelin debris, inflammatory responses from non-neuronal cells, and subsequent axonal regeneration. The immune response, recognized for its role in clearing myelin debris and modulating the local inflammatory milieu, is crucial for initiating axonal regeneration at the proximal stump of nerves. Nevertheless, the precise mechanisms by which the immune response influences PNI and the strategies to harness this process to augment regeneration remain elusive. This article provides a comprehensive overview of the diverse roles and mechanisms of the immune system in PNR and presents insights into potential therapeutic strategies. Furthermore, the article examines immune-associated signaling pathways and their impact on PNR, underscoring the significance of immune modulation in enhancing patient outcomes with PNI. Ultimately, it encapsulates and forecasts the theoretical and practical directions of this field.

## 1 Introduction

The peripheral nervous system (PNS) is predominantly composed of nerve fibers and neuronal cell bodies situated outside the central nervous system (CNS). It functions as a conduit, facilitating communication between the PNS and CNS by transmitting signals through fibers, thereby connecting peripheral receptors to the CNS (1). Nerve injuries are prevalent clinical conditions, frequently associated with motor and sensory impairments, and contribute significantly to disability rates. The incidence of peripheral nerve injury (PNI) resulting from diverse traumatic events is estimated to be between 1.3% and 2.8% (2). Patients with severe injuries frequently encounter the challenge of enduring lifelong disabilities, which impose significant physical suffering and economic strain on both their families and society at large.

Upon reaching maturity, mammals exhibit a distinct difference in regenerative capacity between the CNS and PNS, with the latter retaining a notable ability to regenerate. In the event of PNI, spontaneous regeneration is initiated, driven by the activation of the immune microenvironment and the intrinsic growth potential of neurons. This regenerative process encompasses several key stages, including distal axon fragmentation, myelin sheath degeneration, clearance of myelin debris, inflammatory responses from non-neuronal cells, and axonal regrowth (3). It is well established that the immune response plays a crucial role in facilitating axonal regeneration at the proximal site by clearing myelin debris and modulating the local inflammatory milieu. However, the specific mechanism of the immune response during PNI and how to utilize it to improve peripheral nerve regeneration (PNR) remain a challenge. This article provides a summarization of various roles and mechanisms played by the immune system during the process of PNR, and offers insights into the prospects and potential treatment strategies for it.

## 2 The process of regeneration after PNI

The regeneration process following PNI involves a sequence of well-defined steps. Initially, PNI results in damage to the cytoplasmic membrane of the affected neuronal cells, causing a substantial influx of calcium (Ca^2+^) and sodium (Na^+^) ions and the subsequent generation of action potentials. These action potentials are conveyed retrogradely to the neuronal cell body, prompting the neurons and proximal axons to initiate preparations for growth (4). To support axonal regeneration, Wallerian degeneration occurs in the distal part of the injured nerves (5). The peripheral glial cells, supporting cells and Schwann cells, express high levels of the demyelination related genes, promoting Schwann cell dedifferentiation and clear myelin debris, together with macrophages (6). Subsequently, Schwann cells undergo proliferation and assume a reparative phenotype, traversing the basement membrane to establish Büngner bands (7). They secrete neurotrophic factors, including nerve growth factor (NGF), brain-derived neurotrophic factor (BDNF), and neurotrophic factor-3, which facilitate the guidance of growth cones into the contralateral endoneurial tube (8). Subsequently, Schwann cells re-ensheath the nerve axons, maintaining high expression levels of myelination-related genes such as Krox20, PMP22, MAG, and MBP (9), thereby forming mature myelin. Ultimately, the neural tissue reinnervates the target tissue or organ, leading to the restoration of function.

## 3 Immunity and PNR

Upon damage to peripheral nerves, the body’s response is swift, with the immediate activation of the innate immune system. In the initial phase of injury, there is a localized activation of the innate immune response, resulting in the recruitment of leukocytes. Neutrophils are the first to be mobilized, playing a crucial role in the clearance of myelin debris and the mediation of an inflammatory environment (10). Once the debris is thoroughly cleared, T lymphocytes infiltrate the injured sciatic nerve (5). These T lymphocytes modulate the subsequent stages of the immune response by secreting cytokines that can either promote pro-inflammatory or anti-inflammatory pathways, thereby supporting both cellular and humoral immunity. During the anti-inflammatory repair phase, there is a substantial secretion of anti-inflammatory mediators, which facilitate cell proliferation and the regeneration of new tissues (11). Ideally, peripheral nerve injuries undergo a gradual healing process, aided by the immune system. However, if the inflammatory phase is excessively prolonged, it can lead to adverse outcomes, such as chronic wounds, pathological scar formation, and even failure to regenerate (12).

### 3.1 Mechanism of immune regulation in PNR

#### 3.1.1 Regulation of inflammatory response

Inflammation is a natural response to injury, characterized by the recruitment of immune cells to the site of damage. This response is essential for clearing debris and facilitating the regeneration of nerve tissues. In this process, macrophages play a pivotal role in the inflammatory response, transitioning from a pro-inflammatory M1 phenotype to a healing M2 phenotype, which is crucial for promoting nerve repair and regeneration (13). Immune cells regulate the intensity and duration of the inflammatory response by releasing various cytokines and chemokines. For instance, pro-inflammatory factors such as IL-1β and TNF-α are highly expressed during the initial stage of injury, clearing myelin debris and initiating the inflammatory response; whereas anti-inflammatory factors such as IL-10 and TGF-β are upregulated during the later stages of repair, inhibiting excessive inflammatory response and promoting tissue repair (14). Additionally, Treg cells have been identified as important modulators that can suppress excessive inflammatory responses, thereby preventing neuropathic pain and promoting a more favorable environment for nerve regeneration (15).

#### 3.1.2 Regulating axonal regeneration

The interaction between the immune system and neuronal cells is vital for axonal regeneration. For example, the blockade of glial inhibitors, combined with the activation of intrinsic neuronal growth pathways, has been demonstrated to induce axonal regeneration (16). This suggests that targeting both immune responses and neuronal growth factors can synergistically enhance recovery after injury. Moreover, the role of macrophages in modulating the environment for axonal growth is significant. Multipotent adult progenitor cells have been shown to shift macrophages from a pro-inflammatory state to an anti-inflammatory state, which not only prevents axonal dieback but also promotes regrowth after spinal cord injury (17).

Immune cells facilitate axonal regeneration through both direct and indirect mechanisms, primarily by secreting a range of growth factors and extracellular matrix proteins. For example, macrophages secrete neurotrophic factors, including NGF, BDNF, and glial cell line-derived neurotrophic factor (GDNF), which directly enhance axonal growth (18). Furthermore, immune cells contribute to the formation of a supportive scaffold for axonal extension by modulating extracellular matrix components within the local microenvironment, such as fibronectin and laminin (19).

#### 3.1.3 Interaction between the immune system and Schwann cells

The interaction between Schwann cells and immune cells is critical for nerve regeneration. Schwann cells not only provide structural support but also secrete factors that influence macrophage behavior, thereby orchestrating the immune response at the injury site (20). For instance, the secretion of GDNF by Schwann cells can enhance macrophage polarization toward the M2 phenotype, promoting axonal regeneration (21). The dynamic interplay between the immune system and Schwann cells highlights the importance of immune modulation in PNR. Strategies that enhance the recruitment of pro-regenerative macrophages or modulate their polarization could lead to improved outcomes in nerve repair. Understanding these mechanisms provides valuable insights into developing therapeutic interventions aimed at enhancing PNR.

#### 3.1.4 Regulation of neural regeneration through various signaling pathways

The signaling pathways of immune regulation play a crucial role in PNR, orchestrating the complex interactions between various cell types, including Schwann cells, macrophages, and neurons. The involvement of specific signaling pathways, such as the NF-κB/STAT3 cascade, has been highlighted in regulating axonal growth and immune responses post-injury. NF-κB is activated in sensory neurons following peripheral axotomy and is crucial for promoting axon growth (22). Additionally, the mTOR signaling pathway has been shown to be reactivated in Schwann cells during the dedifferentiation phase after nerve injury, which is necessary for myelin clearance and subsequent remyelination (23). Moreover, the PI3K/Akt and ERK/MAPK signaling pathways have been implicated in various aspects of neuron survival and axonal regrowth. These pathways not only support neuronal health but also influence the behavior of Schwann cells and macrophages, thereby modulating the immune environment conducive to regeneration (24).

### 3.2 The role of various immune cells in PNR

**Figure 1:**
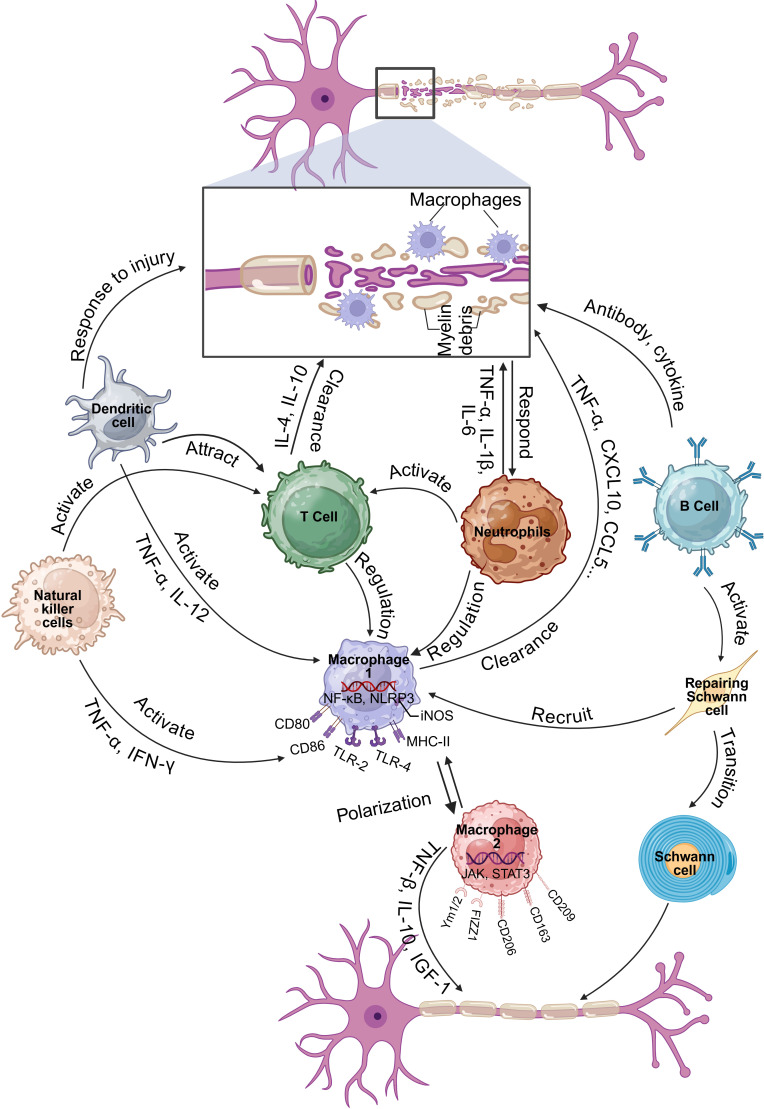
Function and mechanism of immune cells in regulating PNI and PNR (Created in https://BioRender.com).

PNR is a complex process that involves the coordinated interaction of various cell types, including immune cells. The involvement of immune cells in PNR underscores the importance of the immune system in facilitating nerve repair. Understanding the specific roles and interactions of these cells can provide insights into developing targeted therapies to enhance nerve regeneration and improve functional recovery after PNI (**Figure 1**).

#### 3.2.1 Macrophages

Upon PNI, Wallerian degeneration ensues progressively within the first week post-injury. During this period, Schwann cells and damaged axons release soluble factors that activate resident macrophages and attract blood-derived macrophages to the injury sites. In the initial stages following nerve damage, macrophages are swiftly recruited and predominantly exhibit a pro-inflammatory M1 phenotype. These M1 macrophages secrete a range of pro-inflammatory cytokines, including tumor necrosis factor alpha (TNF-α), interleukin-1 beta (IL-1β), and interleukin-1 (IL-6), and are involved in the phagocytosis and degradation of myelin debris and necrotic cells. This process is crucial for clearing the injury site, mitigating the inflammatory response, and creating a favorable microenvironment for subsequent regeneration processes (25). Following the clearance of the injury site and the attenuation of the inflammatory response, macrophages progressively shift toward an anti-inflammatory M2 phenotype (26). M2 macrophages release a range of anti-inflammatory cytokines and growth factors, including interleukin-10 (IL-10), transforming growth factor beta (TGF-β), and insulin-like growth factor-1 (IGF-1), which facilitate tissue repair, extracellular matrix reconstruction, and the promotion of nerve regeneration (27). Throughout the nerve regeneration process, macrophages modulate the local immune milieu by interacting with other immune cells, such as T cells and B cells, thereby maintaining a balance between pro-inflammatory and anti-inflammatory responses (28). Furthermore, macrophages interact with Schwann cells to synergistically promote axonal regeneration and myelin repair, as noted in previous studies (29). Therefore, the macrophage response is deemed essential for the regeneration of injured peripheral nerves.

#### 3.2.2 Neutrophils

Neutrophils, another class of immune cells, rapidly infiltrate the site during the initial phases of nerve injury. They are functional in clearing cellular debris through phagocytosis and contribute to tissue degradation and clearance by releasing a variety of enzymes and reactive oxygen species (ROS). Nonetheless, an excessive accumulation of neutrophils can provoke an overwhelming inflammatory response, potentially leading to detrimental effects (30).

Following nerve injury, neutrophils are among the first immune cells to be recruited to the site of injury. Their rapid arrival is assisted by chemotactic factors, including CXCL1 and CXCL2 (31). Neutrophils release a variety of factors, such as cytokines (e.g., TNF-α, IL-1β, IL-6) and chemokines (e.g., MIP-2) (32), which serve to attract and activate additional immune cells, including macrophages and T cells (33). At the injury sites, the primary role of neutrophils is to clear cellular debris and pathogens through the process of phagocytosis. They degrade and digest cellular debris and pathogens through the release of ROS and enzymes, such as myeloperoxidase (34), thereby facilitating the clearance of the injury site and establishing a conducive environment for subsequent regenerative processes.

Although the primary role of neutrophils is to clear debris and prevent infection, but their activity can also lead to unintended consequences that impede nerve regeneration. One of the adverse effects of neutrophils is their ability to release neutrophil extracellular traps (NETs), which can delay the repair process in Wallerian degeneration by inhibiting macrophage infiltration into the parenchyma. This inhibition is harmful because macrophages play a significant role in clearing myelin debris and facilitating nerve regeneration. The formation of NETs outside the parenchyma acts as a barrier, preventing the necessary macrophage activity that supports nerve repair (33). In addition, neutrophils can release toxic soluble factors, such as matrix metalloproteinases, ROS and cytokines, which have neurotoxicity and thereby aggravate the peripheral injury (35).

#### 3.2.3 T cells

T cells are also integral to the repair of peripheral nerve injuries. Research indicates that T cells are involved in the inflammatory response following nerve injury, which can either enhance or impede regeneration, contingent upon their subtype and the local microenvironment. For example, CD4^+^ T cells and CD8^+^ T cells infiltrate the damaged area. CD4^+^ T cells modulate macrophage polarization and function through the secretion of cytokines, including IL-4 and IL-10, thereby promoting nerve regeneration (15). Conversely, CD8^+^ T cells contribute to the maintenance of tissue integrity by eliminating infected or injured cells in the damaged area via cytotoxic mechanisms (36). Regulatory T (Treg) cells have been identified as pivotal in mitigating neuropathic pain by suppressing the Th1 response at the site of injury (15). This indicates that Treg cells may enhance the environment for nerve regeneration by modulating the immune response.

The presence of activated T cells has also been linked to adverse effects on nerve repair. Empirical evidence suggests that a deficiency in T cells may facilitate enhanced functional recovery post-PNI, as demonstrated in studies utilizing RAG2^-/-^ mice, which are devoid of mature T and B lymphocytes (37). These findings underscore the possibility that T cells may impede motor recovery following nerve injury, indicating that their accumulation could be detrimental in certain contexts. Additionally, the accumulation of T cells within acellular nerve allografts has been shown to be length-dependent, affecting the regenerative capacity of these grafts (38). This indicates that the spatial and temporal dynamics of T cell infiltration are important factors that can dictate the success of nerve repair strategies.

#### 3.2.4 B cells

While traditionally, macrophages and Schwann cells have been the primary focus in studies of PNR, recent findings suggest that B cells also play a significant role in this process. B cells are known for their functions in antibody production and immune regulation, but they may also contribute to nerve repair through the secretion of neurotrophic factors and cytokines that can influence the behavior of other cells involved in regeneration. After PNI, B cells secrete antibodies and cytokines to eliminate pathogens and debris at the injury site, participate in regulating local inflammatory responses, and thereby create a more favorable microenvironment for nerve regeneration (37). B cells can be activated and migrate to the injury site, where they may help modulate the inflammatory response. This modulation is crucial, as inflammation can either promote or inhibit nerve regeneration depending on its nature and timing (39). For instance, a balanced immune response involving both pro-inflammatory and anti-inflammatory signals is necessary for optimal nerve healing (40).

Moreover, the interaction between B cells and Schwann cells may also facilitate nerve regeneration. Schwann cells can respond to signals from B cells, potentially influencing their proliferation and differentiation, which are critical for effective nerve repair (41). In addition, the genetic features of young and aged animals after PNI suggest that the immune response, including B cell activation, varies significantly with age, impacting the overall regeneration capacity (42). Understanding the specific mechanisms by which B cells influence PNR could lead to novel therapeutic strategies aimed at enhancing recovery after nerve injuries.

#### 3.2.5 Natural killer cells

Following PNI, natural killer (NK) cells have been shown to infiltrate the injured site and exert significant effects on the regeneration process. They regulate the local inflammatory response by secreting cytokines (such as Interferon-gamma (IFN-γ), and TNF-α) and chemokines. These cytokines can activate other immune cells, such as macrophages and T cells, thereby forming a coordinated immune response. This enhances the phagocytic activity and antigen-presenting ability of macrophages, thereby improving the response of T cells and aiding in the clearance of debris and pathogens at the injury site (43). It has been demonstrated that NK cells can selectively induce the degeneration of damaged sensory axons through the re-expression of specific ligands in dorsal root ganglion neurons, which triggers their cytotoxic activity (44). This process is crucial as it complements the natural Wallerian degeneration, facilitating the clearance of damaged axons and potentially enhancing the regenerative environment for intact axons.

#### 3.2.6 Dendritic cells

Dendritic cells play a crucial role in the immune response and have been increasingly recognized for their involvement in PNI and PNR. They initiate and regulate the initial immune response by recognizing and ingesting antigens from damaged cells and pathogens. Activated dendritic cells secrete various cytokines and chemokines (such as IL-12 and TNF-α) at the injury site, attracting other immune cells such as macrophages and T cells to the site, thereby enhancing the local immune response (45). Dendritic cells are the most effective antigen-presenting cells in the body. They present the ingested antigens to T cells through MHC class I and MHC class II molecules, thereby activating T cells. In this way, dendritic cells play a bridging role in the regeneration process after neural injury, converting the innate immune response into a specific adaptive immune response (46). Dendritic cells can not only activate pro-inflammatory T cells (such as Th1 and Th17), but also regulate the activity of anti-inflammatory T cells (such as Treg cells) by secreting anti-inflammatory cytokines (such as IL-10 and TGF-β) (43). This dual regulatory role helps balance the local immune response, protecting neural tissue from excessive inflammatory damage while promoting the formation of a regenerative environment.

### 3.3 Immune associated signaling pathways and PNR

**Figure 2:**
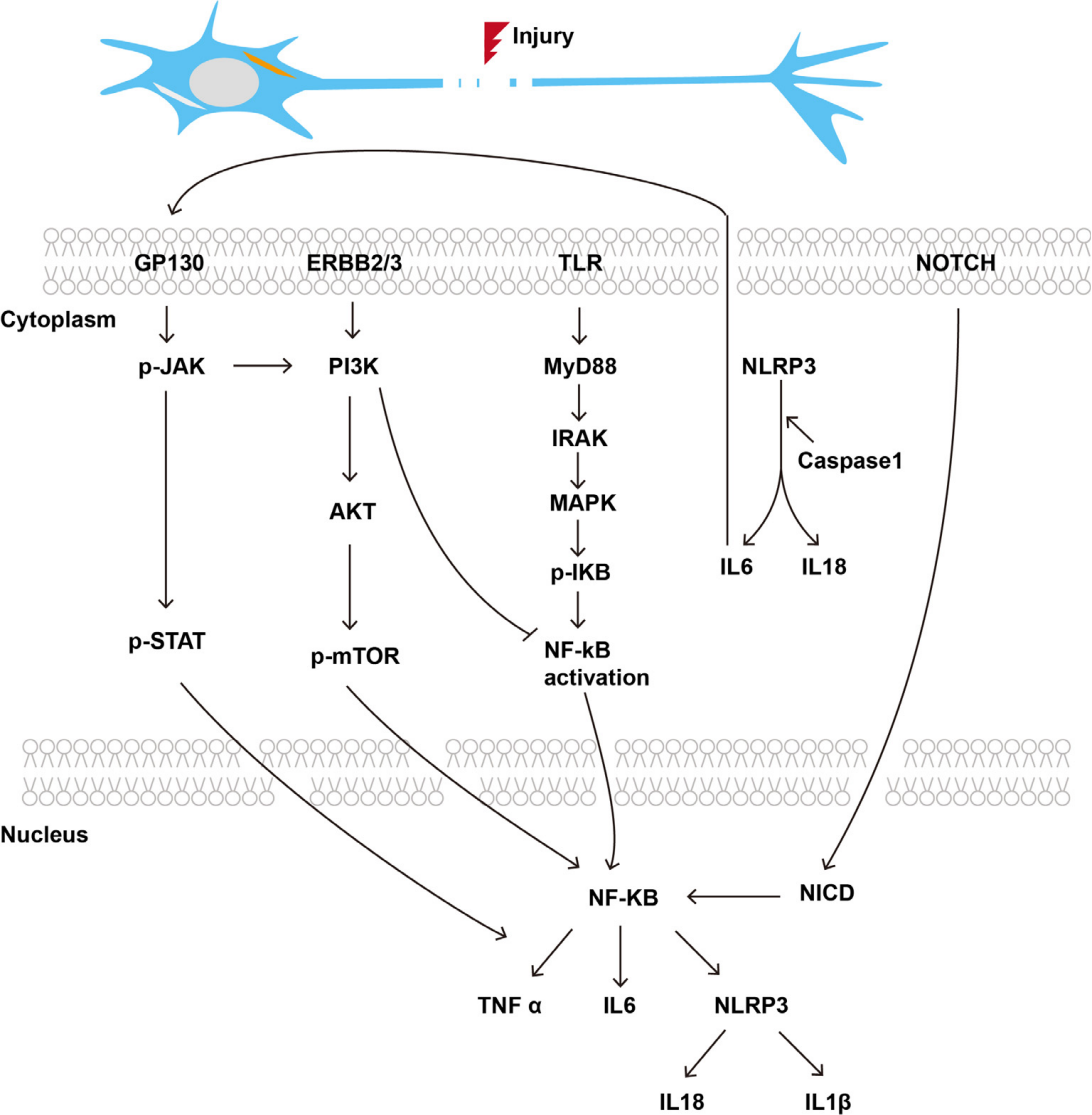
Immune related signaling pathways in the process of PNR.

Immune associated signaling pathways play a crucial role in PNR, influencing both the inflammatory response and the subsequent repair processes. The intricate relationship between immune signaling pathways and PNR is critical for understanding the mechanisms underlying nerve repair. By elucidating these pathways, new therapeutic strategies can be developed to improve outcomes in patients suffering from PNI (**Figure 2**).

#### 3.3.1 Toll-like receptor signaling pathway

Toll-like receptor (TLR) signaling is integral to the orchestration of innate immune responses, facilitating the efficient and rapid removal of inhibitory myelin debris and thereby enhancing PNR (47). The initial expression of interleukin-1β and monocyte chemoattractant protein-1 in the distal stump of the sciatic nerve is impaired in mice with defects in TLR signaling transduction. Conversely, animals administered a single microinjection of TLR2 and TLR4 ligands at the site of sciatic nerve injury demonstrated an accelerated clearance of degenerated myelin sheaths and a reduced recovery period compared to control rats (47). TLRs are activated following PNI, promoting an inflammatory response. Specifically, TLR4 induces the production of inflammatory cytokines, such as TNF-α, IL-1β, and IL-6, by activating the nuclear factor kappa B (NF-κB) and MAPK signaling pathways. These cytokines play a pivotal role in the early inflammatory response, aiding in the clearance of cellular debris and pathogens from the injury sites (48).

#### 3.3.2 NLRP3 inflammasome pathway

The NLRP3 inflammasome is activated following PNI, promoting the maturation and release of IL-1β and IL-18 (49). These cytokines play a role in the nerve regeneration process by regulating the inflammatory response and promoting the survival and growth of nerve cells. Studies have shown that Berberine significantly downregulates the expression of the NLRP3 inflammasome and its related molecules in macrophages, thereby alleviating macrophage M1 polarization and inflammation induced by NLRP3 inflammasome activation (50). Consequently, the neuroprotective effects of Berberine are concomitant with the suppression of inflammation following PNI, primarily through the inhibition of macrophage M1 polarization induced by NLRP3 inflammasome activation (50).

#### 3.3.3 JAK/STAT signaling pathway

PNI elicits distinct expression profiles of growth factors and cytokines, which play a crucial role in modulating the response to injury and facilitating regeneration, through specific intracellular signaling pathways. For instance, the JAK/STAT signaling pathway mediates signal transduction via the GP130 receptor complex, with the activation of signal transducer and activator of transcription (STAT3) being pivotal in modulating the immune cell response at the site of injury (51). PNI also stimulates the production of IL-6-related neurotrophic cytokines, which contribute to neuroprotection and regeneration via the STAT pathway, which is locally activated in damaged nerves (52).

#### 3.3.4 PI3K/Akt/mTOR signaling pathway

The PI3K/Akt signaling pathway is activated by growth factors, including NGF and BDNF, thereby promoting neuronal survival and axonal regeneration. This pathway is essential in regulating cellular growth, proliferation, and survival. Studies have demonstrated that the administration of exogenous glial cell line-derived neurotrophic factor into the buccinator muscle enhances the expression of growth-associated protein 43 and confers neuroprotection to facial neurons via the PI3K/Akt/mTOR signaling cascade. This intervention enhances the regeneration of facial nerves post-crush injury and aids in the restoration of neural conduction function (53). Another study demonstrated that CXCL12 expression was elevated during the initial stages of facial nerve injury and diminishes after two weeks. CXCL12 markedly enhanced Schwann cell migration, decreased the phosphorylation of PI3K, AKT, and mTOR, and elevated the expression of the autophagy marker LC3 II/I. In the animal study, CXCL12 was found to improve facial nerve function and promote myelin regeneration, suggesting its potential as a key therapeutic target (54).

#### 3.3.5 NF-κB signaling pathway

NF-κB signaling pathway plays a pivotal role in regulating the immune system and has significant implications for PNR. It is crucial for the expression of various pro-inflammatory cytokines and mediators that are involved in immune responses. Aberrant activation of NF-κB has been linked to several pathological conditions, including autoimmune diseases and neurodegenerative disorders, which can adversely affect nerve regeneration (55, 56).

In PNI, NF-κB signaling is activated in response to inflammatory stimuli, leading to the production of cytokines such as TNF-α and IL-1β, which can exacerbate neuropathic pain and hinder regeneration (57). Studies have shown that inhibiting NF-κB activity can alleviate pain and improve functional recovery following nerve injuries, suggesting that targeting this pathway may enhance nerve regeneration (58, 59). Moreover, another research indicates that immune modulation through NF-κB signaling can influence the behavior of glial cells in the spinal cord, which are critical for maintaining homeostasis and supporting neuronal health during the regeneration process (60). The interplay between NF-κB and various signaling pathways, including those involving macrophage polarization, further underscores its importance in orchestrating the regenerative response following PNI (61).

#### 3.3.6 Notch signaling pathway

Notch signaling has emerged as a critical pathway in the regulation of various biological processes, including immune responses and tissue regeneration. Notch signaling has been shown to influence macrophage polarization, thereby affecting their role in nerve repair (62). During the axonal injury regeneration process, there is a marked increase in the number of autophagic vesicles. The activation of autophagy facilitates the expedited clearance of myelin debris and enhances the repair process. Previous studies have demonstrated that DLK-mediated injury signaling can activate autophagy, which may subsequently limit the levels of LIN-12 and Notch proteins, thereby promoting axonal regeneration (63). Furthermore, it is well established that dedifferentiated Schwann cells are crucial for neural regeneration, and Notch signaling plays a significant role in activating the proliferation and differentiation of Schwann cells, effectively promoting neural repair in the early regeneration stage (64).

### 3.4 Immunoactive substances

#### 3.4.1 Complement

The complement system plays an important role in the injury and regeneration of the PNS. Complement not only participates in the clearance of pathogens and the regulation of inflammatory response, but also plays a key role in the repair process after nerve injury. Studies have shown that complement components are activated after PNI and promote nerve regeneration by regulating the function and migration of immune cells (65). The signaling of complement C3 was found to be essential for skeletal muscle regeneration, a process that involves the function and migration of monocytes (65). Some components of the complement system, such as C1q and C3, have been shown to promote axonal regeneration of retinal ganglion cells after optic nerve injury. These complement components promote the cleaning of the injury site and the establishment of a regenerative environment through the interaction with microglia and monocytes (66). In treating PNI, the regulation of the complement system may provide opportunities for the development of new therapeutic strategies. For example, treatment targeting the complement system can help improve the effect of nerve regeneration, especially in the case of severe injury (67). By regulating the activity of complement, it may enhance the regeneration ability of Schwann cells, thus promoting the functional recovery of nerves (68).

#### 3.4.2 Cytokines

Cytokines are small protein messengers that serve as critical mediators in various physiological and pathological processes. They are secreted by one cell and can act autocrinely on the same cell or paracrinely on other cells. They play a pivotal role in the immune response, acting as signaling molecules that facilitate communication between cells. Concurrently, they play a role in the pathogenesis of inflammation associated with various diseases (69). Currently, biologic agents targeting cytokines are extensively utilized in therapeutic interventions for tumors, autoimmune diseases, immune deficiencies, infections and other aspects.

##### 3.4.2.1 Interleukin

The role of interleukin (IL) in PNI and regeneration is a critical area of research, as inflammatory cytokines significantly influence the healing process following nerve damage. Interleukins, particularly pro-inflammatory cytokines, are released in response to PNI and play a pivotal role in mediating the inflammatory response that is essential for nerve regeneration. For instance, interleukin-1β (IL-1β) has been shown to be upregulated during the early phases of nerve injury, facilitating the regeneration process through the nuclear factor-κB signaling pathway (70). This cytokine enhances the expression of various neurotrophic factors, thereby promoting neuronal survival and functional recovery. Interleukin-6 (IL-6) is another key player in the regenerative process. Following PNI, IL-6 signaling is activated, which contributes to the pro-regenerative state in dorsal root ganglia (DRG) neurons (71). This signaling pathway is crucial for the activation of growth-associated proteins that facilitate axonal regeneration. Interestingly, the presence of IL-6 has been linked to the conditioning effect observed in DRG neurons, enhancing their regenerative capacity even in non-injured segments (71).

In addition to these pro-inflammatory cytokines, IL-10 serves an important anti-inflammatory role. It is known to downregulate the production of pro-inflammatory cytokines, thus helping to resolve inflammation and promote a favorable environment for nerve repair (72). The balance between pro-inflammatory and anti-inflammatory cytokines is vital for optimal nerve regeneration, as excessive inflammation can hinder recovery. Furthermore, the involvement of interleukin-17 (IL-17) in neuropathic pain following PNI has been documented. IL-17 contributes to neuroinflammation and pain hypersensitivity, indicating that while some interleukins promote regeneration, others may exacerbate pain and hinder recovery (73). This dual role underscores the complexity of cytokine signaling in the context of nerve injury and regeneration.

##### 3.4.2.2 Colony stimulating factor

Colony stimulating factor (CSF), particularly granulocyte-colony stimulating factor (G-CSF) and macrophage colony-stimulating factor (M-CSF), have been shown to play significant roles in modulating the immune response and promoting the survival and proliferation of nerve cells and supporting cells, such as Schwann cells, during the regeneration process. Studies have indicated that G-CSF possesses neurotrophic properties that can enhance nerve regeneration following PNI. In experimental models, G-CSF treatment has been associated with improved functional recovery and increased numbers of regenerating axons (74). The granulocyte-macrophage colony-stimulating factor (GM-CSF) has been shown to exert positive effects to stimulate early axonal regeneration after the PNI (75).

##### 3.4.2.3 Interferon

Interferon (IFN) plays essential roles in PNI and PNR as it intersects with various immune responses and cellular mechanisms that facilitate nerve repair. IFN-γ, is known to modulate immune responses and have been implicated in the regulation of inflammation following PNI. The inflammatory response is essential for clearing debris and creating a conducive environment for regeneration, but it must be tightly regulated to avoid excessive damage to the nerve tissue (39). IFN-γ also enhances the M1 macrophage response, which can initially help in clearing debris and pathogens but may also lead to detrimental effects if the inflammatory response is excessive or prolonged (76). Furthermore, the use of IFN-γ in therapeutic strategies for enhancing nerve regeneration has been explored. For example, the administration of IFN-γ in conjunction with other growth factors has shown promise in preclinical models, suggesting that a combined approach may yield better outcomes in nerve repair (77, 78).

##### 3.4.2.4 Tumor necrosis factor

The role of tumor necrosis factor (TNF) in PNI and regeneration is multifaceted, involving both detrimental and beneficial effects. TNF is a pro-inflammatory cytokine that plays a crucial role in the inflammatory response following PNI. Elevated levels of TNF have been associated with increased pain sensitivity and the development of neuropathic pain, as seen in studies where TNF inhibition resulted in reduced mechanical allodynia and improved recovery outcomes in various animal models of nerve injury (79–81).

In the context of nerve regeneration, TNF can also exert protective effects. For instance, TNF signaling through its receptors has been shown to influence the survival and differentiation of oligodendrocyte precursor cells, which are essential for remyelination after nerve injury (82). Moreover, TNF can activate pathways that promote axonal growth and regeneration, particularly through its interaction with the NF-κB signaling pathway (83). This duality highlights the complexity of TNF’s role, where it can facilitate recovery under certain conditions while exacerbating injury in others. Additionally, studies have indicated that TNF inhibitors, such as etanercept, can suppress local inflammatory reactions and facilitate recovery in models of olfactory nerve injury, further supporting the potential of TNF modulation in promoting nerve regeneration (84).

In summary, immunoactive substances play multifaceted roles in PNI and PNR, influencing both the inflammatory response and the regenerative capacity of neurons and supporting cells (**Table 1**). Understanding these mechanisms may lead to the development of targeted therapies aimed at enhancing nerve repair and functional recovery.

**Table 1:** Typical studies reported on the immunoactive substances in treating PNI.

Substance	Tissue	Function	Reference
Complement C3, C1q	Skeletal muscle, optic nerve	Promoting skeletal muscle regeneration and axonal regeneration, cleaning of the injury site and the establishment of a regenerative environment	(65, 66)
Interleukin (IL-1β)	Sciatic nerve	Enhancing the expression of various neurotrophic factors, promoting neuronal survival and functional recovery	(70)
Interleukin (IL-6)	Dorsal root ganglia	Facilitating axonal regeneration, activating growth-associated proteins	(71)
Interleukin (IL-10)	Peripheral nerve	Inhibiting inflammation and promoting nerve injury	(72)
Interleukin (IL-17)	Sciatic nerve	Increasing neuroinflammation and pain hypersensitivity, hindering recovery	(73)
Granulocyte-colony stimulating factor	Sciatic nerve	Modulating immune response, promoting the survival and proliferation of nerve cells and supporting cells	(74, 75)
Interferon (IFN-γ)	Sciatic nerve	Modulating immune responses and inflammation, enhancing nerve regeneration	(76–78)
Tumor necrosis factor (TNF)	Sciatic nerve, olfactory nerve, spinal cord	Bad effects: increasing neuropathic pain; good effects: promoting remyelination, axonal growth and regeneration, regulating the survival and differentiation of oligodendrocyte precursor cells	(79–84)

## 4 Strategies for promoting PNR via immune regulation

Integrating strategies that modulate immune responses, whether through biomaterials, pharmacological agents, or a combination of both, holds great potential for enhancing PNR and improving functional recovery following nerve injuries.

### 4.1 Biomaterials and immune cells

The use of biomaterials that can influence the local immune microenvironment has emerged as a promising strategy. Biomaterials play a crucial role in providing structural support and delivering bioactive factors that enhance nerve repair. For instance, nerve guidance conduits (NGCs) made from various biomaterials can facilitate the regeneration of peripheral nerves by mimicking the natural extracellular matrix, promoting cell adhesion, and guiding axonal growth (85). Recent advancements in biomaterials have included the incorporation of topographical features and neurotrophic factors to improve the efficacy of these conduits (77).

The immune system significantly influences the regeneration process. Immune cells, particularly macrophages, are essential for clearing debris and secreting cytokines that promote healing. The polarization of macrophages toward a pro-regenerative M2 phenotype has been shown to enhance nerve regeneration. Studies have demonstrated that biomaterials can modulate the immune response, promoting a favorable environment for nerve repair. For example, nerve guidance conduits made from electrically aligned polyurethane have demonstrated the ability to recruit macrophages and skew their polarization toward the M2 phenotype, thereby enhancing PNR in animal models (13). Similarly, composite hydrogels incorporating platelet-rich plasma have been developed to create a regenerative microenvironment that supports nerve repair by sustaining the release of growth factors that modulate immune responses (86).

In addition to macrophages, other immune cells such as T cells and B cells also contribute to the nerve regeneration process. Moreover, the modulation of the immune response through the use of stem cells, such as mesenchymal stem cells (MSCs), has been explored as a therapeutic strategy (87). MSCs can secrete immunomodulatory factors that promote a favorable environment for nerve regeneration by skewing the immune response toward a more regenerative phenotype (88, 89).

Recent advancements in biomaterials and tissue engineering have also highlighted the potential of combining immune cell therapies with engineered scaffolds to enhance peripheral nerve repair. For instance, nerve guidance conduits that incorporate immune-modulating agents or cells can significantly improve functional recovery in animal models (90, 91). The integration of biomaterials and immune system modulation presents a promising strategy for enhancing PNR. Continued research in this area is essential to develop effective therapies that can be translated into clinical practice, ultimately improving outcomes for patients with peripheral nerve injuries.

### 4.2 Pharmacological approaches

Peripheral nerve injuries (PNIs) often lead to debilitating functional deficits, and traditional repair methods, such as autografts, may not always yield satisfactory outcomes. Consequently, researchers are exploring innovative strategies that leverage pharmacological agents and the immune response to enhance nerve regeneration.

Immune system augmentation strategies, such as the administration of glatiramer acetate, have demonstrated positive effects on PNR. An immune-modulating therapy, utilizing glatiramer acetate, has demonstrated potential in enhancing PNR by augmenting the immune response (92). Metformin, for instance, has been found to promote M2 macrophage polarization through the AMPK/PGC-1α/PPAR-γ signaling pathway, thereby enhancing PNR (93). Another pharmacological approach involves the blockade of glial inhibitors, such as chondroitin sulfate proteoglycans, which are known to impede axonal growth. Combining this blockade with the activation of intrinsic neuronal growth pathways has shown synergistic effects on axonal regeneration (16). This dual approach not only reduces extrinsic inhibition but also enhances the intrinsic capacity of neurons to regenerate. Furthermore, the modulation of autophagy has been identified as a critical factor in promoting nerve regeneration. Studies have indicated that enhancing autophagic processes can lead to improved motor function recovery following nerve injuries, suggesting that pharmacological agents targeting autophagy may represent a viable therapeutic strategy (94). Intersection of drug development, immune system modulation, and PNR presents a promising frontier in therapeutic strategies aimed at improving recovery outcomes following nerve injuries.

## 5 Conclusion and perspective

Immune response may be a significant factor contributing to the difference in regenerative capacity between PNS and CNS. After PNI, the injured site undergoes pro-inflammatory clearance of foreign bodies and anti-inflammatory repair and regeneration stages. The transition between pro-inflammatory and anti-inflammatory stages supports good regeneration of peripheral nerves. Immune cells play multiple roles in PNI repair, including regulating inflammatory response, promoting axonal regeneration, and reconstructing neural connections. Future research should further explore the specific mechanisms of different types of immune cells and how to optimize the repair process after nerve injury by regulating immune response. This will provide an important theoretical basis for developing new treatment strategies.
